# Sociobiome signals by high income for increased mobile genetic elements in the gut microbiome of Chinese individuals

**DOI:** 10.3389/fmicb.2025.1596101

**Published:** 2025-05-26

**Authors:** Chen Tian, Zhigang Zhang

**Affiliations:** State Key Laboratory for Conservation and Utilization of Bio-Resources in Yunnan, School of Life Sciences, Yunnan University, Kunming, China

**Keywords:** mobile genetic elements, public health, microbiome, antibiotic resistance genes, Chinese individuals

## Abstract

**Introduction:**

Mobile genetic elements (MGEs) play a crucial role in the dissemination of antibiotic resistance genes (ARGs), posing significant public health concerns. Despite their importance, the impact of socioeconomic factors on MGEs within the human gut microbiome remains poorly understood.

**Methods:**

We reanalyzed 1,382 publicly available human gut metagenomic datasets from Chinese populations, including 415 individuals from high-income eastern regions and 967 individuals from low- and middle-income western regions. MGEs were identified and categorized into functional groups, and statistical analyses were conducted to assess regional differences and correlations with economic indicators.

**Results:**

A total of 638,097 nonredundant MGEs were identified. Among these, MGEs related to integration/excision had the highest mean abundance, while those involved in stability/transfer/defense had the lowest. The abundance of MGEs was significantly higher in the eastern population compared to the western population. Moreover, MGE abundance was positively correlated with regional GDP per capita and with ARG abundance within individuals.

**Discussion:**

Our findings suggest that socioeconomic development and industrialization are associated with increased MGE abundance in the human gut microbiome, which may in turn facilitate the spread of ARGs. These results highlight a potential unintended consequence of economic advancement on public health through microbiome-mediated antibiotic resistance.

## Introduction

Over the past two centuries, human health has markedly improved, driven in large part by steady income growth and advancements since the Industrial Revolution (Weil, 2014). Higher income for individuals or countries improves health in many ways, such as providing better nutrition and building public health infrastructure (Weil, 2014). However, industrial progress—including the widespread use of antibiotics, the adoption of processed food diets, and increasingly sanitized environments—has significantly influenced the composition and transmission of the human microbiota (Sonnenburg and Sonnenburg, 2019). Recent studies have also suggested that different socioeconomic statuses or income levels may affect human health by influencing the gut microbiota (Kwak et al., 2024; Zuniga-Chaves et al., 2023; Harrison and Taren, 2018), which may open new avenues for creating more equitable health outcomes across all socioeconomic levels. The gut microbiota plays a pivotal role in maintaining host health (Kuziel and Rakoff-Nahoum, 2022) through mobile genetic elements (MGEs), which serve as key facilitators of horizontal gene transfer (HGT) and enable genetic exchange within microbial communities (Forster et al., 2022). Notably, MGEs have significant public health implications because of their critical role in the dissemination of antibiotic resistance genes (ARGs) (Forster et al., 2022). However, to date, how socioeconomic factors affect the variation in MGEs in the human gut microbiome remains unexplored.

China, as a country characterized by pronounced regional disparities (Yang et al., 2024), presents significant differences between its eastern and western regions in terms of lifestyle, health care standards, and industrialization levels. The eastern region, driven by rapid economic development, is generally associated with increased urbanization, increased consumption of industrialized food, and a more robust health care system. However, these advancements may be accompanied by issues such as increased antibiotic consumption (Zhang et al., 2015) and environmental pollution. In contrast, the western region has experienced relatively slow economic growth, and traditional dietary and lifestyle practices are more prevalent, with differing patterns of antibiotic usage and health care resource allocation. Our previous study revealed that industrialization, as reflected by economic growth, has led to a decrease in the diversity of the gut microbiota and ARGs in the Chinese population (Tian et al., 2025). Thus, it is necessary to investigate the associations between socioeconomic factors and the dynamic changes in MGEs of the human gut microbiome between Eastern China and Western China to develop more scientific public health infrastructure.

## Results

**Figure 1:**
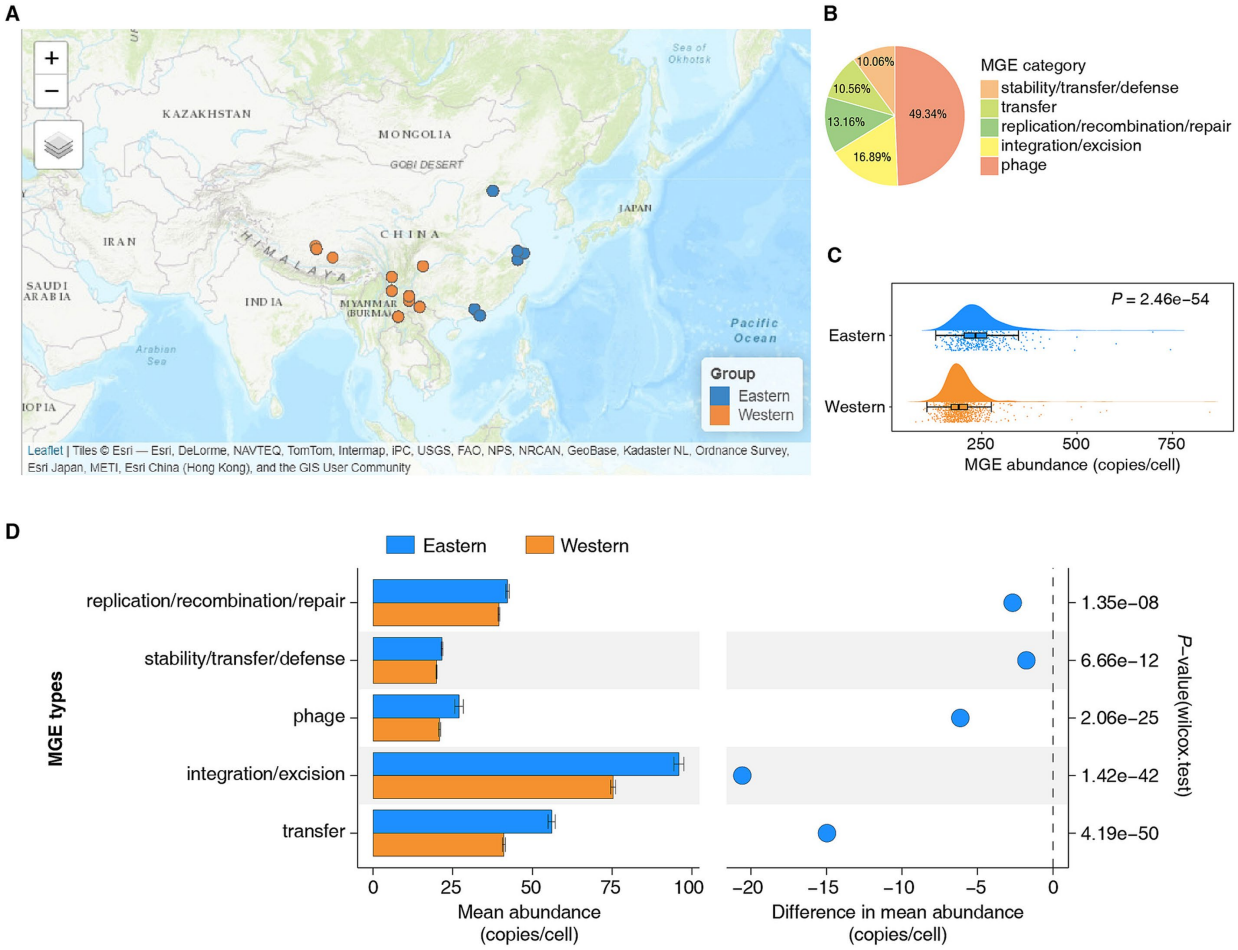
Comparative analysis of the abundance of mobile genetic elements in the gut microbiota of Eastern and Western Chinese populations. **(A)** Sampling distribution map. The blue dots represent the Eastern Chinese populations, whereas the orange dots represent the Western Chinese populations. Refer to Supplementary Table 1 for comprehensive details. **(B)** Categories of MGEs in the gut microbiota of the Chinese population. **(C)** The abundance of MGEs in the gut microbiota of Eastern populations was significantly greater than that in Western populations (Wilcoxon rank sum test, *p* = 2.46e−54). **(D)** Statistical analysis and comparison of the mean abundance of different categories of MGEs in the gut microbiota of Eastern and Western populations.

To elucidate the dynamics of MGEs in the human gut microbiome, we reanalyzed 1,382 human gut metagenomic datasets from the Chinese population, including 415 individuals from the high-income eastern region and 967 individuals from the low- and middle-income western region (Figure 1A; Supplementary Table 1). Previous studies have demonstrated that this dataset reliably represents the gut microbiota characteristics of healthy Chinese populations from these regions (Tian et al., 2025). The batch effect within the dataset is minimal, and there are no significant differences in age distribution between the samples from the eastern and western regions (Tian et al., 2025). Using the ARG-OAP (v3.2.4) (Yin et al., 2023) pipeline and the mobileOG-db (Release: beatrix-1.6) (Brown et al., 2022) database, we annotated MGEs on clean reads. A total of 638,097 nonredundant MGEs were identified, which were primarily classified into five categories on the basis of their essential functions (Figure 1B; Supplementary Table 2). Among them, MGEs in the phage category were the most abundant (Figure 1B). To ensure comparability across different samples, we normalized the abundance of nonredundant MGEs in each sample using copies per cell as the unit.

The results revealed that the abundance of MGEs in the gut microbiota of the Eastern population was significantly greater than that in the Western population (Figure 1C, Wilcoxon rank sum test, *p* = 2.46e−54). Given that the gut microbiota is influenced by a variety of factors, we stratified the comparison of MGE abundance in the gut microbiota of the Eastern and Western populations by sex, ethnicity, and lifestyle. The results consistently indicated that the abundance of MGEs in the gut microbiota of the Eastern population was significantly greater than that in the Western population, regardless of sex, ethnicity, or lifestyle (Supplementary Figures 1A–C, Wilcoxon rank sum test, ***p < 0.001, ****p < 0.0001). Additionally, we categorized and quantified the abundance of MGEs in the gut microbiota of both Eastern and Western populations. The results revealed that, regardless of region, the integration/excision category of MGEs had the highest mean abundance, whereas the stability/transfer/defense category had the lowest mean abundance (Figure 1D). Notably, the mean abundance of each MGE category in the gut microbiome of the Eastern population was greater than that in the Western population (Figure 1D; Supplementary Tables 3, 4, Wilcoxon rank sum test, *p* < 0.05).

**Figure 2:**
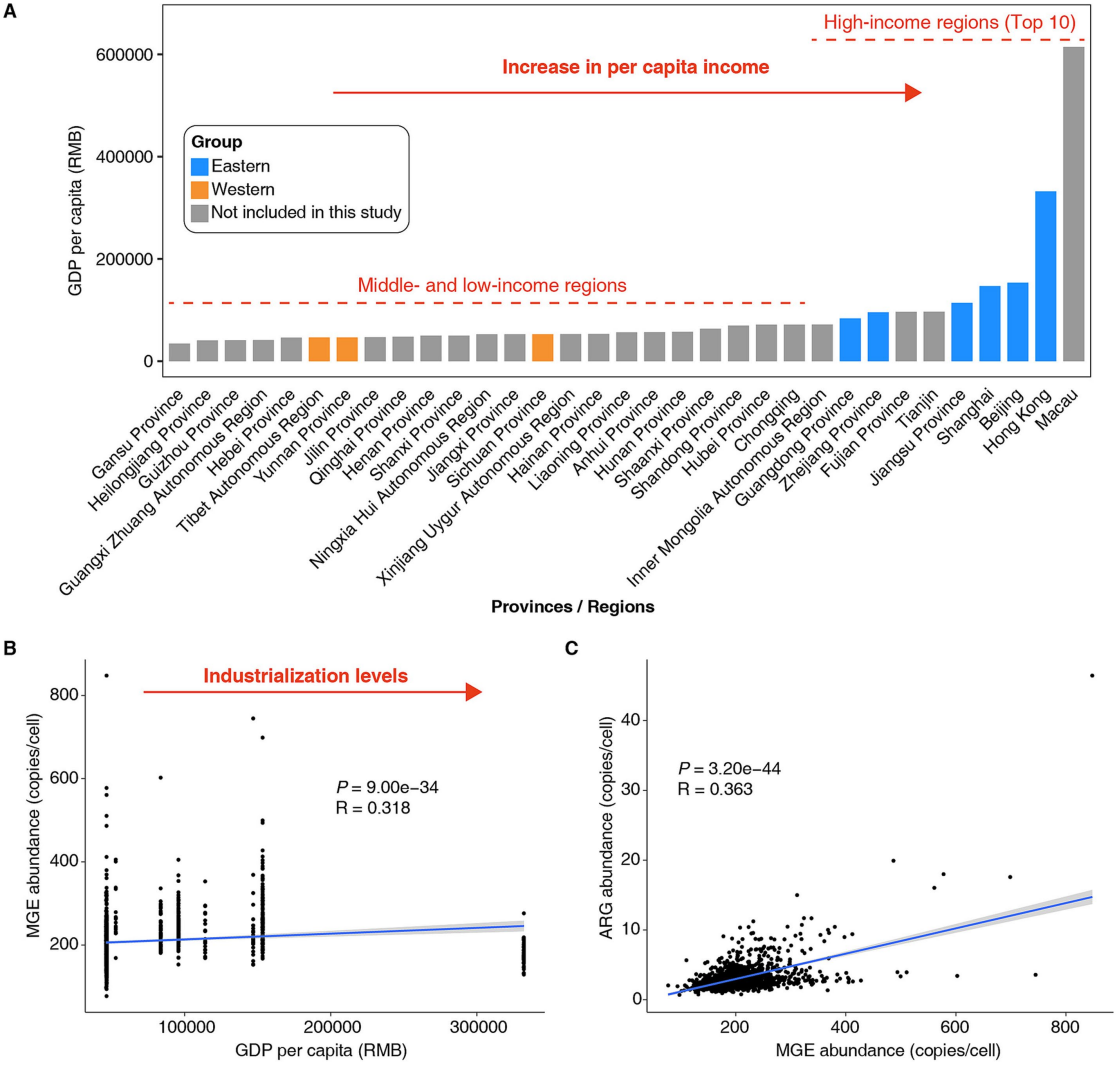
Association between the abundance of MGEs in the human gut microbiome and GDP per capita in China. **(A)** Statistical data on GDP per capita in different provinces/regions of China over the past 10 years. Data were sourced from the publicly available statistics of the National Bureau of Statistics of China (https://data.stats.gov.cn/). In this study, the top 10 provinces/regions in China ranked by per capita GDP will be defined as high-income regions, while the others will be classified as middle- and low-income regions. **(B)** There was a positive correlation between the abundance of MGEs in the gut microbiota of the Chinese population and GDP per capita. **(C)** There was a positive correlation between the abundance of ARGs and MGEs in the gut microbiota of the Chinese population. Spearman’s rank correlation coefficient (R) and statistical significance (*P*) were calculated. Each dot represents a sample, and light gray areas represent their 95% confidence intervals.

The abundant MGEs in the gut microbiome may increase the likelihood of HGT events (Horne et al., 2023). Previous studies have shown that industrialized populations experience a relatively high frequency of HGT in their gut microbiome (Groussin et al., 2021). In general, industrialization is synonymous with economic development and increasing per capita income (Jiejiao and Qin, 2021). Further analysis based on data from the National Bureau of Statistics of China^1^ revealed that, in the past decade, the GDP per capita of the Western populations in this study ranked relatively low among different provinces/regions in China, whereas the GDP per capita of the Eastern populations ranked in the top 10 (Figure 2A). We identified a positive correlation between GDP per capita and the abundance of MGEs in the gut microbiota of Chinese populations (Figure 2B, *R* = 0.318, *p* = 9.00e−34). In other words, the level of industrialization may drive the increase in MGE abundance in the gut microbiota of Chinese populations. Furthermore, we observed a positive correlation between the abundance of MGEs and ARGs in individual gut microbiomes (Figure 2C, *R* = 0.363, *p* = 3.20e−44). Moreover, our previous study revealed a positive correlation between the number of MGEs and the number of ARGs in bacterial genomes (Tian et al., 2025). These findings suggest that MGEs may serve as vehicles for ARG transfer within the gut microbiome, potentially increasing microbial resistance to antibiotics.

## Discussion

Previous studies have confirmed that the ARG burden in soil and urban sewage in Eastern China is significantly greater than that in Western China (Zheng et al., 2022; Su et al., 2017). However, our previous study revealed that the abundance and diversity of ARGs in the gut microbiota of Eastern Chinese populations were significantly lower than those in Western populations. We speculate that the high abundance of MGEs in the gut microbiomes of individuals from Eastern populations may be related to environmental pressures. Environmental selection is one of the main factors influencing the abundance of MGEs (Zhang et al., 2022). Rapid economic development in Eastern China, marked by increased industrialization, urbanization, and increased antibiotic usage, has promoted the selection of bacteria carrying ARGs, which are mostly modulated by MGEs such as plasmids, transposons, and integrons (Forster et al., 2022). Economic growth has reduced the gut antibiotic resistome in the Chinese population but has increased the abundance of MGEs in individual gut microbiota. This finding is of particular concern from a public health perspective, as it suggests that economic development and industrialization, while improving overall health outcomes in certain regions, may inadvertently facilitate the spread of antibiotic resistance. In turn, increased antibiotic resistance poses significant challenges for public health systems, particularly in regions experiencing rapid economic growth and increased antibiotic consumption.

Several constraints of our study should be noted. First, MGE annotation was performed directly on unassembled short-read metagenomic data, which are highly fragmented and preclude recovery of complete element backbones. As a result, we classified MGEs by their encoded functional modules (e.g., integrases, transposases) using the mobileOG-db (Brown et al., 2022) resource rather than by full backbone sequences; backbone-based classification—requiring long-read or assembly-based approaches—would offer greater phylogenetic resolution and more precise element typing. Second, antibiotic usage can markedly influence gut microbiota composition and MGE abundance (Li et al., 2023), but detailed province-level antibiotic consumption data are not publicly available. Therefore, we used GDP per capita as a broad proxy for socioeconomic status—given its correlation with healthcare infrastructure and antibiotic access (Klein et al., 2018)—but acknowledge that direct measures of antibiotic use, stewardship policies, and healthcare availability would likely provide a more accurate explanation of regional MGE variation. Finally, the geographic scope of the analysis was limited by the availability of suitable metagenomic datasets from only a few provinces. Although we observed a statistically significant association between GDP and total MGE abundance (R^2^ ≈ 0.1), this modest correlation indicates that economic status alone does not fully account for the observed variability. Other environmental and lifestyle factors (Zhang et al., 2022)—such as local ecology, agricultural practices, and community behaviors—are likely to contribute substantially to regional MGE profiles. Future studies incorporating long-read sequencing, comprehensive antibiotic usage data, and broader geographic sampling will be essential to address these limitations.

## Materials and methods

### Chinese metagenomic datasets

Detailed information and classification of the 1,382 metagenomic datasets from healthy Chinese individuals used in this study can be found in the publication by Tian et al. (2025).

### Metagenomic quality filtering

The raw reads from 1,382 metagenomic samples were processed using fastp (v0.23.0) (Chen et al., 2018) for adapter trimming and quality filtering with default parameters. To remove host contamination, the host genome index was constructed based on the human genome (RefSeq assembly accession: GCF_000001405.40) and reads mapping to the host were filtered out using Bowtie 2 (v2.5.1) (Langmead and Salzberg, 2012).

### Mobile genetic elements annotation and quantification

MGE identification was performed on the clean metagenomic reads of each sample using ARGs-OAP (v3.2.4) (Yin et al., 2023), leveraging a customized database and structural file from mobileOG-db (Release: beatrix-1.6) (Brown et al., 2022). MobileOG-db (Brown et al., 2022) is a curated and structured core MGE database that integrates over 10 million proteins from eight established MGE-related databases (Liu et al., 2019; Douarre et al., 2020; Pruitt et al., 2007; Camarillo-Guerrero et al., 2021; Grazziotin et al., 2016; Siguier et al., 2006; Leplae et al., 2010; Jiang et al., 2019) and organizes them into orthologous groups based on key MGE-associated functions such as integration, replication, and conjugation. This function-based strategy enabled accurate detection of MGEs even in short-read datasets. We applied the recommended parameters—80% similarity cut-off, 75% query length coverage, and an *e*-value threshold of 1 × 10^−7^—to ensure both high precision and sensitivity (Yin et al., 2023). The relative abundance of MGEs was quantified in universal units of copies per cell, normalized against the cell counts in the analyzed metagenome. The abundance of ARGs can be obtained from the publication by Tian et al. (2025).

### Statistical analysis

The R software (v4.2.1) was employed to perform statistical analyses and graphical representations. The R package tmap (v3.3.4)^2^ was used to visualize the geographical coordinates of the samples on the Esri.WorldTopoMap. Wilcoxon rank sum test was used to evaluate differences in various variables between the eastern and western populations. Spearman correlation analysis was used to assess the relationships between variables in the samples.

## Data Availability

The original contributions presented in the study are included in the article/Supplementary material, further inquiries can be directed to the corresponding author.

## Footnotes

^1^ https://data.stats.gov.cn/

^2^ https://github.com/r-tmap/tmap
