# Supplementary figures and images for: Sociobiome signals by high income for increased mobile genetic elements in the gut microbiome of Chinese individuals
Source: Front Microbiol. 2025 May 26;16:1596101. doi: 10.3389/fmicb.2025.1596101 (PMC12146312; doi:10.3389/fmicb.2025.1596101)

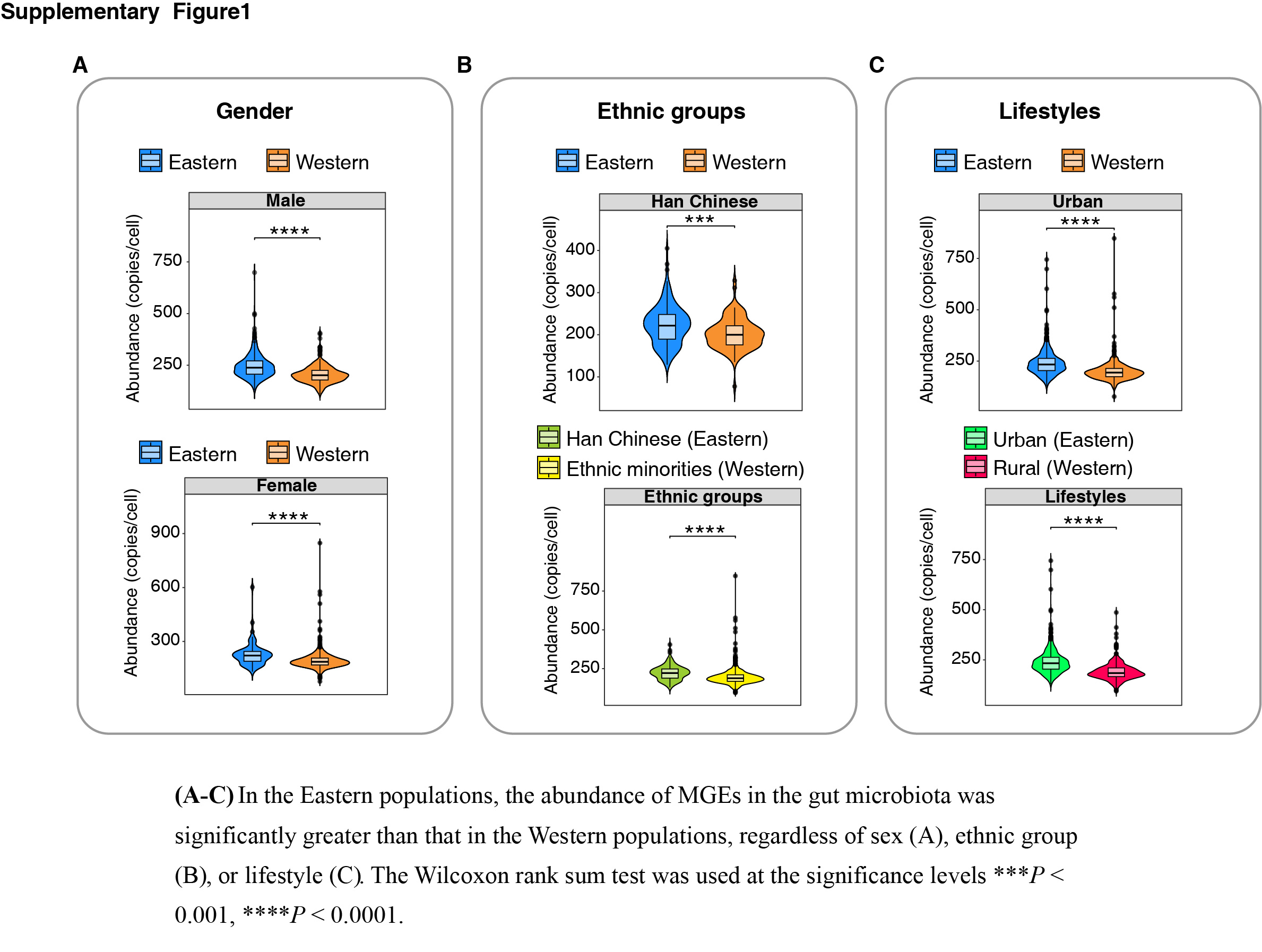

Supplement: Supplementary file 1 [file Image_1.jpeg]
